# Projections of Climate Change Impact on Acute Heat Illnesses in Taiwan: Case-Crossover Study

**DOI:** 10.2196/57948

**Published:** 2024-10-16

**Authors:** Hsiao-Yu Yang, Chang-Fu Wu, Kun-Hsien Tsai

**Affiliations:** 1 Institute of Environmental and Occupational Health Sciences, National Taiwan University College of Public Health, No 17 Xuzhou Road, Taipei, 100, Taiwan, 886 233668102; 2 Department of Public Health, National Taiwan University College of Public Health, Taipei, Taiwan; 3 Population Health Research Center (PHRC), National Taiwan University, Taipei, Taiwan; 4 Department of Environmental and Occupational Medicine, National Taiwan University Hospital, Taipei, Taiwan; 5 Department of Community and Family Medicine, National Taiwan University Hospital Yunlin Branch, Yunlin, Taiwan; 6 Department of Entomology, College of Bioresources and Agriculture, National Taiwan University, Taipei, Taiwan

**Keywords:** climate change, global warming, heat-related illness, carbon reduction, heat, heat illness, extreme heat, Taiwan, real time, epidemic, surveillance, public health, emergency department, early warning system, nonlinear model, temperature, socioeconomic, environmental health, heat stress, environmental, epidemiology

## Abstract

**Background:**

With global warming, the number of days with extreme heat is expected to increase and may cause more acute heat illnesses. While decreasing emissions may mitigate the climate impacts, its effectiveness in reducing acute heat illnesses remains uncertain. Taiwan has established a real-time epidemic surveillance and early warning system to monitor acute heat illnesses since January 1, 2011. Predicting the number of acute heat illnesses requires forecasting temperature changes that are influenced by adaptation policies.

**Objective:**

The aim of this study was to estimate the changes in the number of acute heat illnesses under different adaptation policies.

**Methods:**

We obtained the numbers of acute heat illnesses in Taiwan from January 2011 to July 2023 using emergency department visit data from the real-time epidemic surveillance and early warning system. We used segmented linear regression to identify the join point as a nonoptimal temperature threshold. We projected the temperature distribution and excess acute heat illnesses through the end of the century when Taiwan adopts the “Sustainability (shared socioeconomic pathways 1‐2.6 [SSP1-2.6]),” “Middle of the road (SSP2-4.5),” “Regional rivalry (SSP3-7.0),” and “Fossil-fueled development (SSP5-8.5)” scenarios. Distributed lag nonlinear models were used to analyze the attributable number (AN) and attributable fraction (AF) of acute heat illnesses caused by nonoptimal temperature.

**Results:**

We enrolled a total of 28,661 patients with a mean age of 44.5 (SD 15.3) years up to July 2023, of whom 21,619 (75.4%) were male patients. The nonoptimal temperature was 27 °C. The relative risk of acute heat illnesses with a 1-degree increase in mean temperature was 1.71 (95% CI 1.63-1.79). In the SSP5-8.5 worst-case scenario, the mean temperature was projected to rise by +5.8 °C (SD 0.26), with the AN and AF of acute heat illnesses above nonoptimal temperature being 19,021 (95% CI 2249‐35,792) and 89.9% (95% CI 89.3%‐90.5%) by 2090‐2099. However, if Taiwan adopts the Sustainability SSP1-2.6 scenario, the AN and AF of acute heat illnesses due to nonoptimal temperature will be reduced to 12,468 (95% CI 3233‐21,704) and 62.1% (95% CI 61.2‐63.1).

**Conclusions:**

Adopting sustainable development policies can help mitigate the risk of acute heat illnesses caused by global warming.

## Introduction

### Background

With increasing global warming, the health effects of heat have become an important public health issue. Some studies have reported the relationship between hot temperature and cardiorespiratory disease [1,2], kidney disease [3,4], and mental disorders [5]. An association between heat waves and heat-related illness was also reported in China [6].

Taiwan is an island country in Southeast Asia (23° 58′ N, 120° 58′ E) with a population of 2.3 million. Taiwan’s climate is hot and humid, and the summer begins in June and ends in September. From 1911 to 2005, Taiwan’s temperature warmed by 1.4 °C, indicating that warming in Taiwan is occurring approximately twice as fast as that in the Northern Hemisphere (0.7 °C) [7]. In response to global warming, Taiwan has established a real-time epidemic surveillance and early warning system to monitor acute heat illnesses since January 1, 2011 [8]. Instead of reporting past cases of acute heat illnesses, projecting their future number of acute heat illnesses is crucial for developing prevention strategies and health service planning [9,10]. However, predicting the number of acute heat illnesses requires forecasting temperature changes that are influenced by adaptation policies. To fill this gap, it is crucial to develop methods that use forecasted temperature distributions to estimate the number of acute heat illnesses under various adaptation policies. The Intergovernmental Panel on Climate Change’s Sixth Assessment Report uses 5 shared socioeconomic pathways (SSPs) to take more possible adaptation scenarios into account when modeling future climate change [11]. Global temperature is very likely to increase up to 2.4-4.8 °C in the high-emissions SSP5-8.5 (high-level fossil fuel use) scenario [12].

The Taiwan Earth System Model version 1 (TaiESM1) is a regional climate model specifically designed to simulate and project climate features in Taiwan. Because of the constraint of computing power, the spatial resolution of the earth system models participating in the Coupled Model Intercomparison Project Phase 5 is typically about 100 km [13]. However, this coarse resolution is unsuitable for climate studies in the Taiwan area because this island is 400 km long and 150 km wide and occupies only several grid boxes in these earth system models. The TaiESM1 model can capture local climate features with high resolution, which is crucial for understanding the complex terrain and diverse climatic conditions in Taiwan, such as the influence of the Central Mountain Range in the middle of Taiwan. The model incorporates detailed topographic data and regional characteristics, allowing it to simulate the unique climate patterns of the island’s weather [14].

### Objective

This study aims to estimate the number of acute heat illnesses in Taiwan for both the past period (2010-2019) and the future period (2090-2099) under different adaptation policies.

## Methods

### Study Design

A 2-stage approach was applied. First, we used a distributed lag nonlinear model (DLNM) to explore the nonlinear and lag effects of temperatures (mean) on emergency room visits for acute heat illnesses [15,16]. Second, depending on different SSP scenarios, we used the model to estimate the number of acute heat illnesses through the end of the 21st century.

### Number of Acute Heat Illnesses

We obtained the daily number of acute heat illnesses from the real-time epidemic surveillance and early warning system of Taiwan. The data are publicly provided by the Centers for Disease Control and Prevention of the Ministry of Health and Welfare. We obtained the number of emergency department visits for acute heat illnesses from January 2011 to July 2023 at the first aid responsibility hospitals in Taiwan. The inclusion criteria for acute heat illnesses included (1) heat and light effects (*International Classification of Diseases, Ninth Revision, Clinical Modification* [*ICD-9-CM*] code 992), heat stroke (992.0), heat syncope (992.1), heat cramps (992.2), heat exhaustion, anhidrosis (992.3), heat exhaustion due to salt depletion (992.4), heat exhaustion, unspecified (992.5), transient heat fatigue (992.6), heat edema (992.7), and other specific heat effects (992.8); and (2) accidents caused by excessive heat (E900), accidents caused by excessive heat due to weather conditions (E900.0), accidents due to excessive heat of manmade origin (E900.1), and accidents due to excessive heat of unspecified origin (E900.9).

### Climate Data

We obtained the historical daily meteorological data on maximum, mean, and minimum temperatures and relative humidity from the Taiwan meteorological stations of the Central Weather Bureau in Taiwan [17]. The Central Weather Bureau provided 24-hour data from 25 real-time weather monitoring stations in Taiwan. The meteorological data date back to 1960 [18]. The projected temperature was obtained from the Taiwan Climate Change Projection Information and Adaptation Knowledge Platform [19].

### Determination of the Nonoptimal Temperature Threshold for Acute Heat Illnesses

We used segmented linear regression to identify the join point at which there was a significant change in the number of patients [20]. The formula of segmental regression can be expressed as follows:


(1) $$\tau^{*}=\arg{min}_{\tau }\left(\left(\sum_{i=1}^{n_{1}}{\left(y_{i}-\left(\alpha_{1}+\beta_{1}x_{i}\right)\right)}^{2}+\sum_{i=n_{1}+1}^{n}\left(y_{i}-\left(\alpha_{2}+\beta_{2}x_{i}\right)\right)\right)\right)$$

Where τ* is the number of optimal breakpoints, *n* is the total number of data points, *n*_1_ is the number of data points in the first segment (*x*_*i*_≤τ), α_1_ and β_1_ are the parameters for the first segment, and α_2_ and β_2_ are the parameters for the second segment.

To find the breakpoint τ that minimizes the sum of squared residuals (SSRs) for the piecewise regression model, we used numerical optimization techniques such as grid search, where we tested various candidate values for τ and chose the one that minimizes the SSR. This process requires iteratively fitting the piecewise regression model and calculating the SSR for each candidate breakpoint.

We identified the join point as a nonoptimal temperature threshold and used it as a reference value for estimating the relative risk (RR) of acute heat illnesses [21]. The steps to determine the join point were as follows:

1. Visualize the data: begin by creating a scatter plot of data. Look for any patterns, trends, or possible breakpoints that might suggest the existence of distinct segments.
2. Fit the initial model: start by fitting a simple nonlinear regression model to the data. This provides a baseline understanding of the overall relationship between the variables.
3. Consider potential join points: based on the scatter plot, we identified potential join points where the scatter plot showed an inflection point.
4. Fit the segmented model: use a segmented regression model to fit the data. This model consists of multiple linear segments, each with its slope and intercept. The segmented model identifies the join point where the segments shift.
5. Evaluate model fit: examine the goodness of fit of the segmented model compared to the initial linear regression. Check the Akaike information criterion to see if the segmented model provides a better fit to the data.
6. Interpret coefficients: analyze the coefficients of the segmented model to understand the slopes and intercepts of the different segments. The join point corresponds to the value of the independent variable where the transition between segments occurs.
7. Statistical significance: consider the statistical significance of the join point. If it is statistically significant, it suggests that the change in the relationship between the variables is not due to random variation.
8. Visual confirmation: overlay the segmented regression line on the scatter plot to visually confirm that the join point accurately captures the shift in the relationship (Multimedia Appendix 1).

### Temperature Projection

We applied the TaiESM1 to predict the temperature in Taiwan under different SSPs. The TaiESM1 has been evaluated against observational data and has demonstrated good skill in reproducing historical climate variability and trends in Taiwan. It accurately simulates temperature, precipitation, wind patterns, and other climatic variables, providing valuable insights into past climate conditions and future projections. The TaiESM generally agrees with observations during the period 1979‐2005. It performs better than the median of Coupled Model Intercomparison Project Phase 5 models [14]. We used 4 scenarios to project the temperature of Taiwan from 2015 to 2100 when it adopts different SSPs, including “Sustainability—Taking the green road (SSP1-2.6),” “Middle of the road (SSP2-4.5),” “Regional rivalry—A rocky road (SSP3-7.0),” and “Fossil-fueled development—Taking the highway (SSP5-8.5)” [22].

### Statistical Analysis

Because the relationship between temperature and its health effects is nonlinear and has lagged effects, we applied DLNM to explore their relationships. Based on the quasi-Akaike information criterion, we selected the natural cubic spline DLNM to model the nonlinear temperature effects and a polynomial function to model the lagged effects [23]. We found that a maximum lag of 7 days, 5 degrees of freedom for the mean temperature, and 4 degrees of freedom for lag produced the best model fit. The formula was expressed as follows:


(2) $$Log(\mu t)=\alpha +\beta T_{t,l}+\;crossbasis({Temp}_{t,l},lag=7,argvar=list(fun=ns),\;df=5,arglag=list(fun=poly,df=4))+{RH}_{t}+\;NS(DOY,4)+NS(time,3)+\eta {DOW}_{t}$$

Where *t* is the day of the observation, μt is the observed daily number of acute heat illnesses on day *t*, T*_t,l_* is a matrix obtained by applying the DLNM to temperature, β is the vector of coefficients for T*_t,l_, l* is the lag days, RH is relative humidity, NS is the natural cubic spline, DOY refers to the day of the year, and DOW*_t_* is a categorical variable for day of the week. We used the minimal morbidity temperature (MMT), which corresponded to the lowest RR of acute heat illnesses, as the reference value to calculate the attributable number (AN) and attributable fraction (AF) caused by nonoptimal temperature [24]:


(3) $${AF}_{x}=1-\exp(-\beta_{x})$$

(4) $${AN}_{x}=n\cdot {AF}_{x}$$

Where *n* denotes the total number of acute heat illnesses, and the parameter β_*x*_ denotes the coefficient of DLNM when MMT is used as the reference temperature. The AF is the proportion of acute heat illness caused by nonoptimal temperature. AFs can be multiplied by the total number of acute heat illnesses to obtain the number of acute heat illnesses caused by nonoptimal temperatures [25].

### Projecting Acute Heat Illnesses Under Climate Change Scenarios

The projected temperatures from 2015 to 2100 in the SSPs scenarios were added to the established model to estimate the AN and AF of acute heat illnesses due to nonoptimal temperature. We compared changes in AN and AF due to nonoptimal temperatures across 4 adaptation scenarios during 2010‐2019 and 2090‐2099.

We used the *forecast* package of R software (R Foundation for Statistical Computing) for time-series forecasting [26], the *segmented* package for the segmental linear regression model [20], and the *dlnm* package for distributed lag nonlinear modeling [27]. A *P* value less than .05 was considered statistically significant.

### Ethical Considerations

The research ethics committee of National Taiwan University approved this study (202305HM147), which uses secondary data analysis and does not require additional consent.

## Results

### Demographic and Climate Characteristics

A total of 28,661 cases were included from January 2011 to July 2023. The demographic characteristics of the patients and the distribution of mean temperatures are summarized in Table 1.

**Figure 1.**
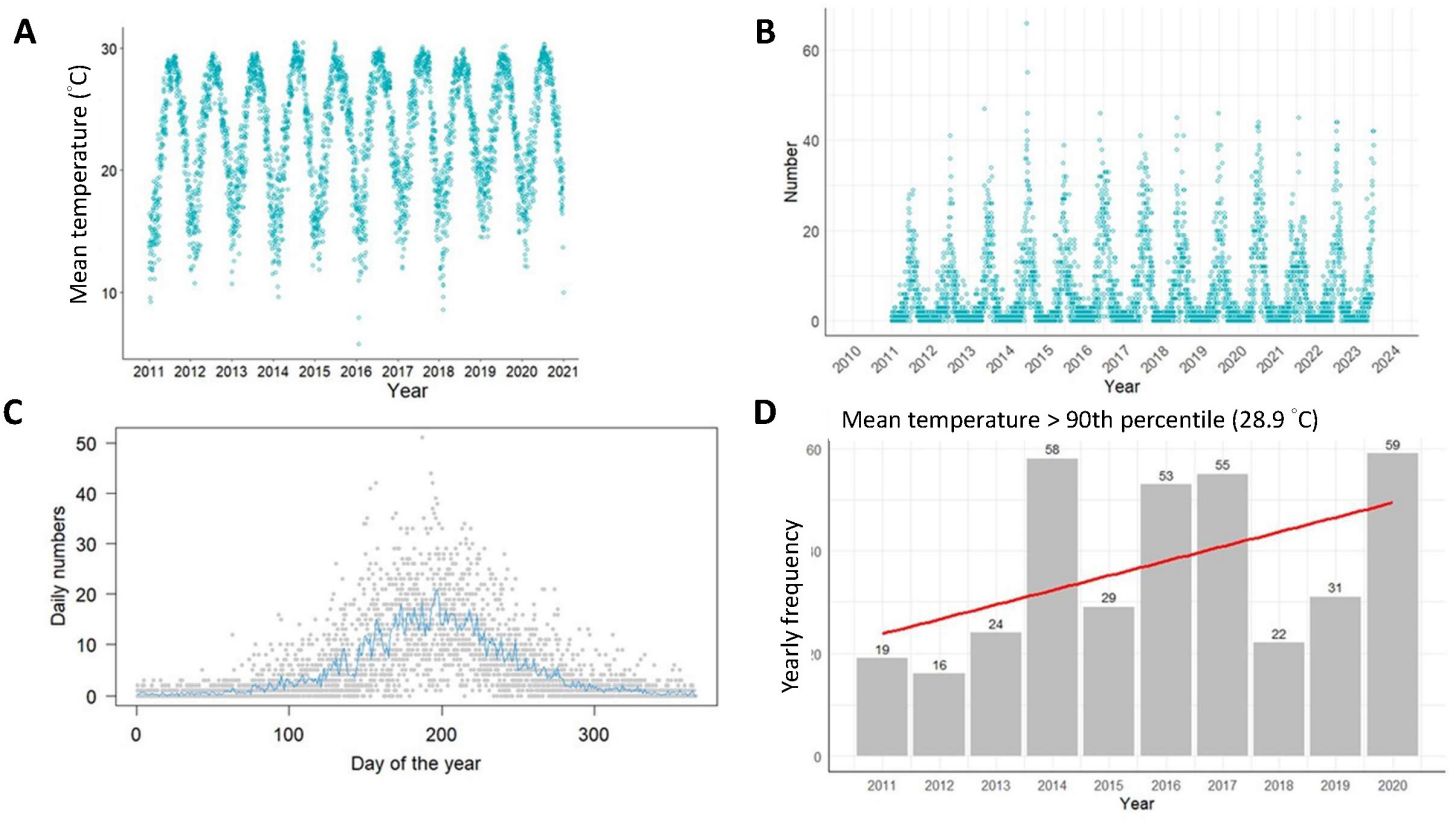
Distribution of temperature, daily number of heat injuries, and trend. (**A**) Scatter plot of temperature. (**B**) Daily number of acute heat illnesses. (**C**) Number of acute heat illnesses per day over the course of a year. The gray spots are the observed daily number of acute heat illnesses, and the blue lines are the daily average acute heat illnesses. (**D**) Number of days when the daily mean temperature exceeded the 90th percentile of the mean temperature.

There was a clear seasonal pattern in temperature in Taiwan (Figure 1A). The mean temperature was 23.2 °C (SD 4.9 °C). A clear seasonal fluctuation of acute heat illnesses was also observed (Figure 1B). Most cases occurred between the 150th and 250th days of the year, which is the summer season in Taiwan (Figure 1C). There is an increasing trend of extreme heat temperature in Taiwan (Figure 1D).

**Table 1.** Demographics of patients and weather distribution in Taiwan during 2011‐2023 (N=28,661).

	Values
**Biological sex, n (%)**	
Male	21,619 (75.4)
**Age (years), mean (SD)**	44.5 (15.3)
**Age group (years), n (%)**	
0‐64	23,628 (82.4)
65‐74	2457 (8.6)
≥75	2576 (9)
**Temperature (°C)**	
Mean (SD)	23.2 (4.9)
Range	5.8-30.5
Median (IQR)	24 (8.3)
90th	28.9
99th	29.8
**Relative humidity (%)**	
Mean (SD)	78.9 (5.6)
Range	63.5-94.6
Median (IQR)	79.2 (7)

### Determination of Nonoptimal Temperature

**Figure 2.**
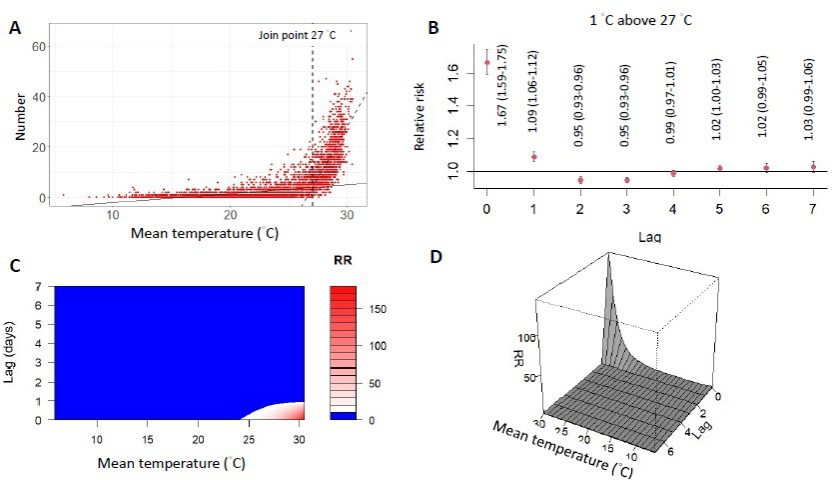
The threshold of nonoptimal temperature and effect of acute heat injuries. (**A**) The temperature-disease relationships in the shape of a “J” with the join point at 27 °C of the mean temperature. (**B**) The lag-response curve of 1 °C above the threshold (27 °C). (**C**) Contour map showing the exposure-lag-response relationship between temperature and acute heat illnesses. (**D**) 3D plot showing the estimated exposure-lag-response association between temperature and acute heat illnesses. RR: relative risk.

By segmented linear regression (Multimedia Appendix 1), the join point of the nonoptimal temperature was 27 °C (Figure 2A). When we used the 27 °C as the threshold value, a 1 °C increase in mean temperature was associated with a 71% increase in acute heat illnesses (RR 1.71, 95% CI 1.63-1.79; Figure 2B). The contour map and 3D plot show that the effect of high temperature on acute heat illnesses was rapid and urgent, with a short lag time within 1 day and a high RR (Figure 2C and D).

### Projection of Mean Temperature

**Figure 3.**
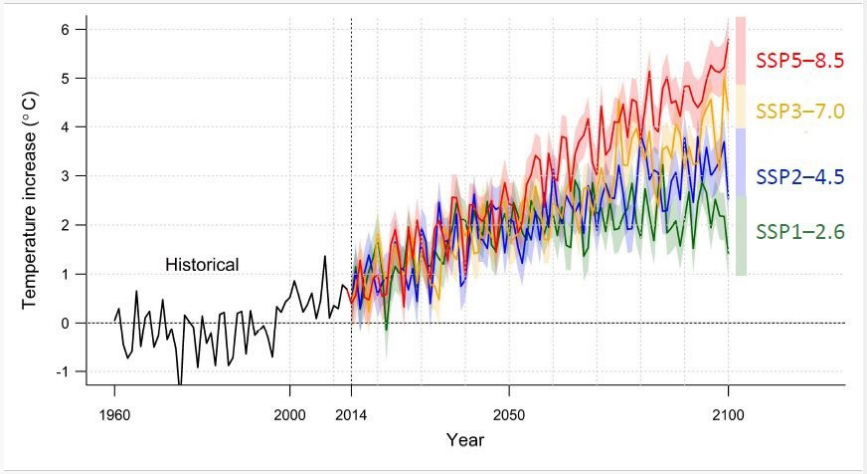
Projection of increased temperature in Taiwan under different climate models. The black line is the historical mean temperature of Taiwan from 1960 to 2014. The red, orange, blue, and green lines are the projected mean temperatures of Taiwan from 2015 to 2099 under different scenarios. Notably, the increase in temperature will be attenuated if Taiwan adopts a sustainability approach (SSP1-2.6). SSP: shared socioeconomic pathway.

Figure 3 shows the projected mean temperature in Taiwan by the end of the century. Under the SSP5-8.5 scenario, the increase in the mean temperature is expected to be +5.8 °C (95% CI 5.3‐6.3). For the SSP3-7.0 scenario, the increase is +4.3 °C (95% CI 3.8‐4.8). Under the SSP2-4.5 scenario, the increase is +2.5 °C (95% CI 2.1‐3.0), and for the SSP1-2.6 scenario, it is +1.4 °C (95% CI 0.9‐1.9).

### Projections of Acute Heat Illness AN and AF

**Figure 4.**
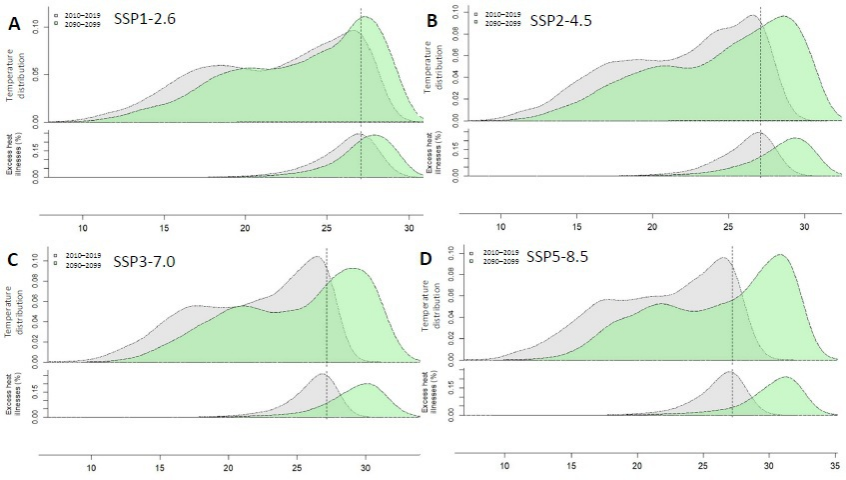
Projections of temperature distribution and excess acute heat illnesses under 4 adaptation scenarios (A) SSP1-2.6, (B) SSP 2-4.5, (C) SSP3-7.0, and (D) SSP5-8.5. The top of each panel shows the temperature distribution, and the bottom of each panel shows the distribution of excess acute heat illnesses expressed as the fraction of additional cases (%) attributed to nonoptimal temperature compared with the minimal morbidity temperature (15.5 °C). The gray area is the period of 2010‐2019, and the green area is 2090‐2099. The vertical dashed line on the right is the threshold of nonoptimal temperature (27 °C). SSP: shared socioeconomic pathway.

Figure 4 shows the distribution of temperature and excess acute heat illnesses at the beginning of the century (2010‐2019) and at the end of the century (2090‐2099). In the SSP5-8.5 worst-case scenario, the AN and AF of acute heat illnesses above nonoptimal temperature (27 °C) was 19,021 (95% CI 2249‐35,792) and 89.9% (95% CI 89.3%‐90.5%) by 2090‐2099. However, if Taiwan adopts the Sustainability SSP1-2.6 scenario, the AN and AF of acute heat illnesses due to nonoptimal temperature (27 °C) will be reduced to 12,468 (95% CI 3233‐21,704) and 62.1% (95% CI 61.2‐63.1; Table 2).

**Table 2.** Attributable number (AN) and attributable fraction (AF) of excess acute heat illnesses above the nonoptimal temperature threshold (27 °C) in different scenarios.

Scenario	Days exceeding nonoptimal temperature (95% CI)		AN of acute heat illnesses exceeding nonoptimal temperature (95% CI)		AF of acute heat illnesses above nonoptimal temperature (%) (95% CI)	
	2010‐2019	2090‐2099	2010‐2019	2090‐2099	2010‐2019	2090‐2099
SSP1-2.6^a^	416 (379-453)	919 (867-969)	6463 (1664-11,262)	12,468 (3233-21,704)	34.2 (33.3‐35.2)	62.1 (61.2‐63.1)
SSP2-4.5	371 (336-408)	1295 (1238-1351)	6021 (1344-10,699)	15,995 (3887-28,102)	31.9 (31‐32.8)	77.6 (76.8‐78.5)
SSP3-7.0	323 (291-358)	1509 (1452-1567)	5198 (904-9492)	17,548 (3546-31,549)	27.6 (26.7‐28.5)	84.3 (83.6‐85)
SSP5-8.5	377 (341-413)	1783 (1725-1840)	6200 (150-12,249)	19,021 (2249-35,792)	32.9 (32‐33.9)	89.9 (89.3‐90.5)

a SSP: shared socioeconomic pathway.

## Discussion

### Principal Findings

This study shows that temperatures stabilize by the end of the century in the sustainability development scenario, with the lowest burden of acute heat illness above nonoptimal temperatures. In response to the impact of global warming, Taiwan is formulating a climate policy aimed at reducing carbon emissions. Our study contributes valuable insights to climate health research, underscoring the importance of proactive measures to address health risks associated with rising temperatures.

This study is valuable, as it develops a new method for estimating nonoptimal temperature join points, thus providing a more reasonable threshold for acute heat illnesses. Different from the temperature-mortality relationship, which is often U-shaped [28], the relationship between temperature and acute heat illnesses is J-shaped with the majority of cases distributed on the left side of the MMT. Since inappropriate temperature thresholds can lead to overestimation or underestimation of disease risk [29], there is a need to develop a new method to determine temperature thresholds [8]. This study shows that the segmented regression is suitable for estimating the threshold temperature for acute heat illnesses, thus providing a more reasonable threshold for acute heat illnesses. However, its suitability depends on the shape of the relationship between the health outcome and temperature. If the relationship is a traditional U shape, where both high and low temperatures increase the risk, segmented regression might not be the best approach. In such cases, other methods might be more effective to capture the nonlinear relationship, such as polynomial regression or spline models.

Our study indicates that the Taiwanese population is more vulnerable to acute heat illnesses compared to other countries. In Taiwan, a 1-degree increase in mean temperature increases the risk of acute heat illnesses by 71% (RR 1.71, 95% CI 1.63-1.79), which is higher than the 18% increase reported in previous meta-analyses (RR 1.18, 95% CI 1.16‐1.19) [30]. Though this difference might be related to the use of different reference temperatures, we should consider the specific vulnerable factors in Taiwan. Taiwan is facing an aging society, and older people are particularly vulnerable to acute heat illness due to multiple existing health conditions [31,32]. We suspect that the aging population might be a significant factor contributing to the higher risk, and we suggest that further studies explore the accessibility of medical care and evaluate the home environments of older people to identify behavioral or environmental factors. Since the vulnerable factors might vary across different cities and countries [33], actions to prevent heat-related health impacts must include identifying these factors in each community [34]. Each identified factor should be considered when designing plans to mitigate vulnerability. A simple heat-related illness screening tool might be useful in identifying individuals at risk [31].

In Taiwan, the government announced the Climate Change Response Act in 2023, which requires all ministries and local governments to assess the impacts of climate change and take practical actions to increase the adaptive capacity of vulnerable groups. This study used data on reported acute heat illnesses from the real-time epidemic surveillance and early warning system of the Ministry of Health and Welfare. Using the data, epidemiologists can quickly estimate trends and risks of diseases associated with global warming, providing timely recommendations for government decision-making.

The use of health surveillance for climate health research and policy can provide timely and actionable information for public health interventions. It can help estimate health impacts on populations, assess the effectiveness of interventions, and inform decision-making to mitigate the health impacts of climate change. The generalizability of our findings to other countries or regions depends on multiple factors, including the availability and quality of health surveillance data, local climatic conditions, and the implementation of climate mitigation strategies. As the threat of heat to public health becomes more apparent, the promotion of national heat hazard prevention strategies to reduce public health impacts is important for policy-making. Spain implemented a Heat Health Prevention Plan between 2004 and 2013. As a result of the program, the impact of temperature on mortality declined. The reduction in mortality due to extreme heat was greater in the provinces where more heat health prevention program measures were implemented [35]. We recommend that the government develop effective strategies for dealing with rising temperatures based on epidemiological evidence.

### Limitations

The real-time epidemic surveillance and early warning system did not cover all hospitals in Taiwan, so there may be underreporting of acute heat-related injuries. The actual number of acute heat illnesses would be higher than our estimate. However, our study used a case-crossover design, which involved comparing the numbers of cases in a time-series sequence. The noninclusion of all hospitals would not influence the association between temperature and the number of acute heat illnesses. In a study of reporting systems, reporting rates may change over time, leading to an increase in reporting in later years. We added a time variable to the DLNM to adjust for this effect, which we conservatively assume would not confound our results. Risk factors for acute heat illnesses include urban residence, age, socioeconomic factors, ethnicity, and race [36], and our data did not include this information. We recommend that future studies consider incorporating additional risk factors, such as air pollution or other interacting variables, and model adaptation scenarios more comprehensively in the future.

### Conclusions

This study estimated the burden of acute heat illness under different adaptation policies. If Taiwan adopts high emissions and limited efforts to mitigate climate change, acute heat illnesses due to nonoptimal temperatures will increase substantially in the future, while the adoption of sustainable development policies can help reduce the risk.

## Supplementary material

10.2196/57948 Multimedia Appendix 1 Estimated coefficient in segmented regression with SE and *P* value.

## Abbreviations

AF: attributable fraction
AN: attributable number
DLNM: distributed lag nonlinear model
*ICD-9-CM*: *International Classification of Diseases, Ninth Revision, Clinical Modification*
MMT: minimal morbidity temperature
RR: relative risk
SSP: shared socioeconomic pathway
SSR: sum of squared residual
TaiESM1: Taiwan Earth System Model version 1
