# Supplementary material for: Projections of Climate Change Impact on Acute Heat Illnesses in Taiwan: Case-Crossover Study
Source: JMIR Public Health Surveill. 2024 Oct 16;10:e57948. doi: 10.2196/57948 (PMC11617331; doi:10.2196/57948)
Supplement: Multimedia Appendix 1 [file publichealth-v10-e57948-s001.docx]

| Interpretation | Estimate | SE | t-value | P-value |
| --- | --- | --- | --- | --- |
| Intercept | -5.46 | 0.45 | -12.18 | <0.05 |
| Mean temperature | 0.35 | 0.02 | 16.59 | <0.05 |
| breakpoint 1*Mean temperature | 7.71 | 0.17 | 45.39 | NA |

Adjusted r-squared 0.74; root mean squared error (RMSE) 4.33.

Estimated break-point 1: 27.04, standard error 0.04.
